# Clinical and radiographic characteristics, management and short-term outcomes of patients with COVID-19 in Wenzhou, China

**DOI:** 10.1186/s12879-020-05528-z

**Published:** 2020-11-13

**Authors:** Liang Hong, Enling Ye, Gangqiang Sun, Xiaoyang Wang, Shengguo Zhang, Yanghe Wu, Xiangao Xie, Shichun Xia, Xudong Zheng, Ling Dong, Fujing Cai, Xixian Lou, Renguo Zhao, Yongqi Hu, Zhanwei Ruan, Jiguang Ding, Qingfeng Sun

**Affiliations:** 1 Department of Infectious diseases, the Third Affiliated Hospital to Wenzhou Medical University (Ruian People’s Hospital), 108 Wansong Road, Ruian, Wenzhou, 325200 Zhejiang Province China; 2 Endocrinology Department, the Third Affiliated Hospital to Wenzhou Medical University (Ruian People’s Hospital), Ruian City, Zhejiang Province 325200 China; 3 Department of Biology, Gordon College, Wenham, MA 01984 USA; 4 Radiography Department, the Third Affiliated Hospital to Wenzhou Medical University (Ruian People’s Hospital), Ruian City, Zhejiang Province 325200 China; 5 Health bureau of Ruian City, 333 Ruihu Road, Ruian, Wenzhou, 325200 Zhejiang Province China; 6 Hospital Office, the Third Affiliated Hospital to Wenzhou Medical University (Ruian People’s Hospital), Ruian City, Zhejiang Province 325200 China; 7 The Emergency Department, the Third Affiliated Hospital to Wenzhou Medical University (Ruian People’s Hospital), Ruian City, Zhejiang Province 325200 China; 8 Pneumology Department, the Third Affiliated Hospital to Wenzhou Medical University (Ruian People’s Hospital), Ruian City, Zhejiang Province 325200 China

**Keywords:** COVID-19, Clinical, Radiographic characteristics, SARS-cov-2, Epidemiology

## Abstract

**Background:**

Coronavirus disease 2019 (COVID-19) is an emerging viral disease. Here, we report the clinical features, management, and short-term outcomes of COVID-19 patients in Wenzhou, China, an area outside Wuhan.

**Methods:**

Patients admitted to the Infectious Diseases Department of Ruian People’s Hospital in Wenzhou, from January 21 to February 7, 2020, were recruited. Medical data on epidemiological history, demographics, clinical characteristics, laboratory tests, chest computerized tomography (CT) examination, treatment, and short-term outcomes were retrospectively reviewed. Blood biochemistry and routine tests were examined using standard methods and automatic machines. CT examination was performed several times during hospitalization as necessary.

**Results:**

A total of 67 confirmed COVID-19 cases were diagnosed; 64 (95.4%) were common cases and three (4.5%) were severe cases. The most common symptoms at admission were fever (86.6%), cough (77.6%), productive cough (52.2%), chest distress (17.9%), and sore throat (11.9%), followed by diarrhea (7.4%), headache (7.4%), shortness of breath (6.0%), dizziness (4.5%), muscular soreness (4.5%), and running nose (4.5%). Thirty patients (47.8%) had increased C-reactive protein levels. The CT radiographs at admission showed abnormal findings in 54 (80.6%) patients. The patients were treated mainly by oxygen therapy and antiviral drugs. By March 3, 2020, all 67 patients completely recovered and had negative nucleic acid tests. The patients were discharged from the hospital and transferred to a medical observation isolation center for further observation.

**Conclusion:**

Cases of COVID-19 in Wenzhou are milder and have a better prognosis, compared to those in Wuhan. Timely and appropriate screening, diagnosis, and treatment are the key to achieve good outcomes.

## Key points

Sixty-four of 67 patients with COVID-19 were considered common cases. By February 17, 2020, 54 patients were discharged and others, including three severe cases, significantly improved. The information is useful for the diagnosis and management of COVID-19.

## Background

Coronavirus disease 2019 (COVID-19) was first reported in Wuhan, Hubei Province, China. The virus, once called 2019 novel coronavirus, was defined by the Coronavirus Study Group of the International Committee as a severe acute respiratory syndrome coronavirus 2 (SARS-CoV-2) on February 7, 2020 [1]. A modeling study reported by Wu et al. stated that as the virus was no longer contained within Wuhan, it could spread to other major cities in China or even worldwide [2]. By February 17, 2020, the date on which we were preparing this report, the number of confirmed cases reached over 70,000 with more than 1700 deaths worldwide; predominately within Wuhan, Hubei province (https://news.qq.com/zt2020/page/feiyan.htm).

The earliest academic papers on the clinical characteristics of COVID-19 were published in late January by the *Lancet* [3, 4]. Up to 99 cases in Wuhan were reported with features of clustering onset, high rates of intensive care unit (ICU) admissions, and a high mortality ranging from 11 to 15% [3, 4]. On January 23, 2020, the World Health Organization estimated the death rate to be 4% [5]. On February 11, 2020, the death rate was 2.9% in Hubei province according to the Novel Coronavirus Pneumonia Emergency Response Epidemiology Team of China Center for Disease Control (CDC) [6]. However, there was no comprehensive description or analysis on the clinical characteristics, management and prognosis of the COVID-19 cases outside the city of Wuhan.

Wenzhou is a commercial hub in Zhejiang Province, China. It is the most-affected city outside Hubei Province, with 503 confirmed cases as of February 17, 2020 (https://news.qq.com/zt2020/page/feiyan.htm). These cases included both the “primary” cases from Wuhan, and “secondary” cases infected by “primary” cases. Whether the clinical characteristics of these secondary cases outside Wuhan are different from that of the cases in Wuhan is unknown. Therefore, the objective of this study was to report the clinical features, management, and short-term outcomes of patients with COVID-19 diagnosed in Wenzhou, China.

## Methods

This is a retrospective study. The protocol of this study was approved by the Ethics Committee of the Third affiliated Hospital to Wenzhou Medical University (Ruian People’s Hospital) (No. YJ2020013). Written informed consent to participate was obtained from all patients included in the study.

### Study population

There were approximately 180,000 Wenzhou people working and studying in Wuhan in 2019, and Ruian is a county-level city under the administration of Wenzhou City. According to incomplete statistics, more than 7000 people returned to Ruian from Wuhan prior to the mandatory Wuhan lockdown. All these people were forcibly quarantined by the authorities for 14 days, and those with fever or respiratory symptoms were sent to the fever clinics. Symptomatic patients were tested with a nucleic-acid test via throat mucus specimens collected with cotton swabs or deep-cough sputum. Patients were then quarantined in Ruian People’s Hospital until they were tested negative for over 14 days. If the test was positive, the patient was diagnosed as a confirmed COVID-19 case. All confirmed cases were admitted to the isolation ward of the hospital. Moreover, all close contacts of each infected person were forcibly quarantined and managed as described for those returning from Wuhan, and a nucleic-acid test was used to screen and confirm people with symptoms. Therefore, almost all people who were infected with the virus in Ruian would have been screened, and thus all COVID-19 cases should have been identified. The data of the patients included in this manuscript have been used in other three published papers but on different topics [7–9].

For this retrospective, single-site study, we recruited patients at the Third Affiliated Hospital to Wenzhou Medical University (Ruian People’s Hospital) in Wenzhou, Zhejiang Province, from January 21 to February 7, 2020. Ruian People’s Hospital is a local comprehensive hospital, with about 2008 beds. On January 21, 2020, the hospital was named as a designated COVID-19 hospital. All patients were admitted to the Infectious Diseases Department of the hospital, which provided quarantine wards with up to 90 beds equipped with a fresh air system for the management of COVID-19 patients. Strict infection-control measures were taken following the third edition of Prevention and Control Program for Pneumonia Caused by COVID-19 by the National Health Commission of the People’s Republic of China. (http://www.gov.cn/zhengce/zhengceku/2020-01/29/content_5472893.htm).

### Diagnostic criteria

The following diagnostic criteria were based on the fourth edition of Diagnosis and Treatment program for Pneumonia Caused by COVID-19 by the National Health Commission of the People’s Republic of China. (http://www.gov.cn/zhengce/zhengceku/2020-01/28/content_5472673.htm).

Suspected cases were diagnosed according to epidemiological history and clinical manifestations. Epidemiological history included: 1) travel or residence history in Wuhan or other places with COVID-19 patients within 14 days before the onset of the disease, 2) exposure to people traveling from Wuhan or other places with COVID-19 patients, and experiencing a fever or respiratory symptoms within 14 days before the onset of the disease, and 3) onset cluster nature or epidemiological link to those with COVID-19. Clinical manifestations included the following: 1) fever (axillary temperature of 37.3 °C or higher), 2) typical radiographic findings (multiple small patchy shadows and interstitial changes appear in the early stage, obvious extrapulmonary bands and multiple ground glass infiltrates to both lobes of the lung may further develop in later stages, and rarely, pulmonary consolidation and pleural effusions occur in severe cases), and 3), white blood cell (WBC) count (WBC count is normal or < 3.5 × 10^9^/L, or the lymphocyte count is < 1.1 × 10^9^/L in the early stage of the disease). Suspected cases were defined when any item of the epidemiological history plus two items of the clinical manifestations were met.

Confirmed cases were defined when the suspected case had one of the following etiological evidence: 1), positivity for novel coronavirus nucleic acid as detected by real-time fluorescence-based RT-PCR of respiratory tract specimens, and 2), high homology with the known SARS-CoV-2 as detected by the virus gene sequencing test of respiratory tract specimens. Accordingly, the incubation period was calculated by the days from exposure to symptom onset.

Patients with confirmed COVID-19 were further classified as common, severe and critically ill cases. Common cases were those with a fever and/or respiratory symptoms with or without radiographic signs of pneumonia. Severe cases were those with one of the following: 1) respiratory distress with a respiratory rate more than 30 breaths per minute, 2) peripheral capillary oxygen saturation (SpO_2_) value ≤93%, and 3) partial pressure of oxygen (PaO_2_)/fraction of inspired oxygen (FiO_2_) ≤ 300 mmHg (1 mmHg = 0.133 kPa). Critically ill cases were those with one of the following: 1) respiratory failure, with a need for mechanical ventilation, 2) shock, and 3) complication with other organ failure requiring ICU monitoring and treatment.

In addition, hypoxemia was defined as oxygen saturation (sO_2_) ≤ 93% or an oxygenation index ≤300 mmHg. Leukopenia was defined as a WBC count < 3.5 × 10^9^/L, thrombocytopenia as a platelet count less than 125 × 10^9^/L, and lymphopenia as a lymphocyte count < 1.1 × 10^9^/L.

### Data collection

Medical data of all COVID-19 patients systematically recorded and stored in a network platform specifically established for SARS-CoV-2 infection and COVID-19 were reviewed retrospectively. The data on epidemiological history, demographics, clinical characteristics, laboratory tests, chest computerized tomography (CT) examination, treatment, and short-term outcomes were collected. Blood biochemistry and routine tests were examined using standard methods and automatic machines. CT examination was performed during hospitalization as necessary. Two independent radiologists read and reported on all chest CT radiographs. Samples for SARS-CoV-2 assays (including nasopharyngeal swabs and sputum) were taken and sent to laboratories of the Ruian CDC. Then, all confirmed and suspected cases were reported to the China CDC Disease Direct Reporting System.

### Data analysis

Continuous data are described as mean and standard deviation (SD), and categorical variables are presented as count and percentage. Student’s *t*-test was used to compare continuous variables, and Fisher’s exact test was used to compare categorical variables. To better characterize patients with COVID-19, a multiple logistic regression was performed to identify independent predictors (a *p* value < 0.1 was used for the univariate analyses). All analyses were performed using a statistical software package (Stata, version 12.0; Stata Corp. Texas, USA). A *p* value of < 0.05 was set as statistically significant.

## Results

### Demographic and clinical characteristics of patients

Overall, there was a total of 67 confirmed COVID-19 cases admitted to the Ruian People’s Hospital from January 21 to February 7, 2020. Thirty-six (53.7%) patients were male. Five patients were suspected patients and excluded from this study. The average age was 45.0 ± 15.2 years, ranging from 5 to 72 years. Thirty-four (50.7%) patients once lived in Wuhan (*n* = 31) or other cities of Hubei (*n* = 3) during December 1, 2019 and January 23, 2020. None of the patients were medical staff. The average incubation period was 5.1 ± 2.3 days in 30 patients; the incubation period was not traceable for the other 37 patients. The most common comorbidities were hypertension (16.4%) and diabetes (0.5%). There were only three (4.5%) smokers and four (6.0%) alcohol abusers. One patient was pregnant, and one patient was under immunosuppressive therapy. The period from symptom onset to admission was 3.8 ± 2.3 days (Table 1). The most common symptoms at admission were fever (86.6%) with a mean axillary temperature of 37.3 ± 0.8 °C, cough (77.6%), productive cough (52.2%), chest distress (17.9%), and sore throat (11.9%), followed by diarrhea (7.4%), headache (7.4%), shortness of breath (6.0%), dizziness (4.5%), muscular soreness (4.5%), and running nose (4.5%) (Table 1).

**Table 1:** Characteristics of 67 patients with COVID-19

Characteristic	All patients (***n*** = 67)	Common patients (***n*** = 64)	Severe patients (***n*** = 3)
**Gender**			
Female	31 (46.3)	30 (46.9)	1 (33.3)
Male	36 (53.7)	34 (50,7)	2 (66.7)
**Age (years)**	45.0 ± 15.2	44.9 ± 15.5	48.3 ± 2.5
5 ~ 15	3 (4.5)	3 (4.7)	0
16 ~ 18	1 (1.5)	1 (1.6)	0
19 ~ 45	33 (49.3)	32 (47.8)	1 (33.3)
46 ~ 65	22 (32.8)	20 (29.9)	2 (66.7)
65 ~ 72	8 (11.9)	8 (12.5)	0
**Exposure history**			
Lived in Wuhan*	31 (46.3)	31 (48.4)	0
Lived in other cities in Hubei province	3 (4.5)	2 (3.1)	1 (33.3)
Exposed to patients in Wuhan	16 (23.9)	14 (21.9)	2 (66.7)
Exposed to local patients	15 (22.4)	15 (23.4)	0
Exposed to local carriers	1 (1.5)	1 (1.6)	0
No epidemic history	1 (1.5)	1 (1.6)	0
**Incubation periods (days)#**	5.3 ± 3.0 (*n* = 34)	5.3 ± 3.1 (*n* = 33)	3.5 ± 0.7 (*n* = 1)
**Period from onset to admission (days)**	3.8 ± 2.3	3.8 ± 2.4	4.0 ± 1.7
**Comorbid illness**			
Cardiovascular disease	1 (1.5)	1 (1.6)	0
Digestive system disease	4 (6.0)	4 (6.3)	0
Malignant tumor	1 (1.5)	1 (1.6)	0
Nervous system disease	0	0	0
Respiratory system disease	1 (1.5)	1 (1.6)	0
Kidney disease	0	0	0
Hypertension	11 (16.4)	11 (17.2)	0
Diabetes	7 (10.5)	7 (10.9)	0
Connective tissue disease*	1 (1.5)	0	1 (33.3)
**Smoking**			
No smoking history	62 (92.5)	59 (92.2)	3 (100)
Previously quit smoking	2 (3.0)	2 (3.1)	0
Current smoker	3 (4.5)	3 (4.7)	0
**Alcohol**			
No alcohol abuse history	61 (91.0)	58 (90.6)	3 (100)
Previously quit drinking	2 (3.0)	2 (3.1)	0
Currently abusing alcohol	4 (6.0)	4 (6.2)	0
Immunosuppressive therapy*	1 (1.5)	0	1 (33.3)
**Pregnancy**	1 (1.5)	1 (1.6)	0
**Symptoms**			
Fever	58 (86.6)	55 (85.9)	3 (100)
Headache	2 (3.0)	2 (3.1)	0
Dizziness	3 (4.5)	3 (4.7)	0
Muscular soreness	3 (4.5)	2 (3.1)	1 (33.3)
Running nose	3 (4.5)	3 (4.7)	0
Sore throat	8 (11.9)	8 (12.5)	0
Cough	52 (77.6)	49 (76.6)	3 (100)
Productive cough	35 (52.2)	32 (50.0)	3 (100)
Chest distress*	12 (17.9)	9 (14.1)	3 (100)
Shortness of breath*	4 (6.0)	2 (3.1)	2 (66.7)
Abdominal pain	2 (3.0)	2 (3.1)	0
Diarrhea	5 (7.4)	5 (7.8)	0
Nausea or vomiting	2 (3.0)	2 (3.1)	0
**Axillary temperature (°C)**	37.3 ± 0.8	37.3 ± 0.8	37.5 ± 0.3
**Chest CT consistent with pneumonia (*n*** **= 65)**			
No	13 (19.4)	13 (20.3)	0
Yes	54 (80.6)	51 (79.7)	3 (100)
**Complications**			
Hypoxemia*	3 (4.5)	0	3 (100)

Data are expressed as mean ± standard deviation or number (%), where appropriate

*Abbreviation*: *SD* standard deviation, *CT* computerized tomography

#, available for 34 cases, including 31 common cases and three severe cases

* *p* < 0.05, compared between common and severe cases

There were 64 (95.4%) common cases and three (4.5%) severe cases. Compared to the common cases, the severe cases were less likely to have been exposed to patients from Wuhan (21.9% vs. 66.7%, *p* = 0.037), and more likely to have a connective tissue disease (33.3% vs. 0%, *p* = 0.045), chest distress (100% vs. 14.1%, *p* = 0.005), shortness of breath (66.7% vs. 3.1%, *p* = 0.008), hypoxemia (100% vs. 0%, *p* < 0.001), or undergoing immunosuppressive therapy (33.3% vs. 0%, *p* = 0.045) (Table 1). Noticeably, one of the severe cases had connective tissue disease, ankylosing spondylitis, and was being treated with recombinant human tumor necrosis factor receptor Fc fusion protein (25 mg per month via subcutaneous injection).

At admission, leukopenia occurred in 17 (25.4%) patients, and a decreased neutrophil count (< 1.8 × 10^9^/L) was found in 19 (28.3%) patients. Also, 12 (17.9%) and eight (11.9%) patients presented with lymphopenia and thrombocytopenia, respectively. One (1.5%) patient had an increased WBC count (11.5 × 10^9^/L) (Table 2). In addition, 32 (47.8%) patients had increased C-reactive protein (CRP). Six (9.0%) patients had low partial pressure of oxygen and two (3.0%) had low partial pressure of carbon dioxide (Table 2). Compared to the common cases, the severe cases had a significant increase in the levels of CRP (*p* < 0.001), gamma-glutamyl transpeptidase (GGT) (*p* = 0.025), creatine kinase (CK) (*p* < 0.001), lactate dehydrogenase (LDH) (*p* < 0.001), and activated partial thromboplastin time (aPTT) (*p* = 0.007) (Table 2).

**Table 2:** The baseline laboratory test results of all 67 study patients with COVID-19

Assay (normal range)	All patients (***n*** = 67 or specified)	Common patients (***n*** = 64 or specified)	Severe patients (***n*** = 3 or specified)
ALT (9–50 U/L)	31.0 ± 31.6	30.3 ± 32.0	46.0 ± 17.0
AST (15–40 U/L)	29.0 ± 18.5	28.3 ± 18.6	45.0 ± 6.2
ALP (45–125 U/L)	79.8 ± 43.1	79.8 ± 44.0	79.3 ± 20.1
GGT (10–60 U/L)	37.3 ± 44.8	35.0 ± 42.3	86.7 ± 77.9
ALB (40–55 g/L)	41.5 ± 5.2	41.5 ± 5.3	40.8 ± 1.7
TP (60–85 g/L)	69.6 ± 5.8	69.6 ± 5.8	71.7 ± 5.2
GLO (20–40 g/L)	28.6 ± 3.9	28.5 ± 3.9	30.9 ± 3.7
A/G (1.2–2.4)	1.5 ± 0.2	1.5 ± 0.2	1.3 ± 0.1
TBIL (0–23 μmol/L)	9.1 ± 4.6	9.1 ± 4.6	9.8 ± 4.2
IBIL (1.2–16 μmol/L)	5.3 ± 3.2	5.3 ± 3.2	5.5 ± 3.0
DBIL (0–8 μmol/L)	3.8 ± 1.6	3.8 ± 1.6	4.3 ± 1.2
TBA (0–10 μmol/L)	6.8 ± 14.5	7.0 ± 14.8	3.5 ± 2.1
PALB (0.2–0.43 g/L)	0.2 ± 0.1	0.2 ± 0.1	0.1 ± 0.04
CHE (4.62–11.5 KU/L)	8.8 ± 2.0	8.8 ± 2.0	10.3 ± 1.3
GLU (3.9–6.1 mmol/L)	6.1 ± 1.3	6.1 ± 1.3	6.1 ± 0.7
BUN (3.6–9.5 mmol/L)	3.9 ± 1.0	4.0 ± 1.0	3.6 ± 0.8
CREA (μmol /L)	65.1 ± 11.0	65.3 ± 11.2	62.3 ± 4.9
K^+^ (3.5–5.3 mmol/L)	3.7 ± 0.4	3.8 ± 0.4	3.7 ± 0.2
Na^+^ (137-147 mmol/L)	138.9 ± 2.5	139.0 ± 2.4	136.6 ± 3.4
CK (50–310 U/L)	100.9 ± 115.4	88.8 ± 69.9	359.7 ± 424.5
CK-MB (0–24 U/L)	23.2 ± 19.9	22.6 ± 20.1	35.7 ± 13.3
LDH (120–250 U/L)	223.0 ± 68.4	215.6 ± 59.3	380.3 ± 68.5
TG (0.56–1.7 mmol/L)	1.3 ± 0.7	1.3 ± 0.7	1.46 ± 0.3
CHO (3.1–5.2 mmol/L)	4.2 ± 0.9	4.2 ± 0.9	4.5 ± 0.3
HDL-C (1.03–3.0 mmol/L)	1.0 ± 0.3	1.0 ± 0.3	1.0 ± 0.2
LDL-C (2.1–3.12 mmol/L)	2.7 ± 0.7	2.7 ± 0.8	3.1 ± 0.3
CRP (0-6 mg/L)	12.1 ± 19.3↑	9.8 ± 13.8↑	61.3 ± 50.2↑
PT (10–13.5 s)	11.0 ± 0.7	11.0 ± 0.7	11.0 ± 0.1
PTA (70–140%)	100.4 ± 10.0	100.4 ± 10.3	99.4 ± 1.4
INR (0.85–1.15)	1.0 ± 0.1	1.0 ± 0.1	1.0 ± 0.006
aPTT (22–36 s)	31.8 ± 4.4	31.5 ± 4.1	37.9 ± 8.5
FIB (2.0–4.0 g/L)	3.8 ± 2.6	3.8 ± 2.6	5.0 ± 0.7
CT (14–21 s)	16.9 ± 0.7	16.9 ± 0.7	16.6 ± 0.6
D-Dimer (0–0.5μg/mL)	0.3 ± 0.3	0.3 ± 0.4	0.3 ± 0.1
WBC (3.5–9.5*10^9/L)	4.7 ± 1.8	4.7 ± 1.8	4.4 ± 0.9
Lymphocyte (1.1–3.2*10^9/L)	1.4 ± 0.6	1.5 ± 0.6	0.9 ± 0.3
Neutrophils (1.8–6.3*10^9/L)	2.8 ± 1.4	2.7 ± 1.4	3.3 ± 0.8
RBC (4.3–5.8*10^12/L)	4.5 ± 0.5	4.5 ± 0.5	4.9 ± 0.5
Hgb (130–175 g/L)	135.1 ± 16.9	134.7 ± 17.0	145.0 ± 12.8
HCT (40–50%)	39.4 ± 4.2	39.3 ± 4.2	42.7 ± 2.6
Platelets (125–350*10^9/L)	194.8 ± 71.9	197.4 ± 72.1	139.7 ± 46.5
Blood PH (7.350–7.450)	7.4 ± 0.03(*n* = 62)	7.4 ± 0.03 (*n* = 59)	7.4 ± 0.008
pO_2_ (80–100 mmHg)	105.8 ± 27.8(*n* = 62)	106.6 ± 27.6(*n* = 59) ↑	89.8 ± 33.5
pCO_2_ (35–45 mmHg)	39.2 ± 3.4(*n* = 62)	39.3 ± 3.4(*n* = 59)	36.4 ± 1.2
sO_2_ (91.9–99.0%)	97.4 ± 1.8(*n* = 62)	97.5 ± 1.6(*n* = 59)	94.8 ± 3.7
HCO3^−^(21-27 mmol/L)	24.5 ± 2.0(*n* = 62)	24.5 ± 2.1 (*n* = 59)	23.7 ± 0.4
AG (8–16 mmol/L)	10.9 ± 2.2(*n* = 62)	11.0 ± 2.2 (*n* = 59)	10.5 ± 2.2
Lac (0.5–1.7 mmol/L)	1.7 ± 0.6(*n* = 62)	1.7 ± 0.6(*n* = 59)	1.7 ± 0.6
PCT (< 0.5 ng/ml)	0.2 ± 0.08 (*n* = 56)	0.2 ± 0.08(*n* = 54)	0.24 ± 0 (*n* = 2)
BNP (0–100.0 pg/ml)	12.2 ± 6.4 (*n* = 59)	12.3 ± 6.5 (*n* = 56)	10.0 ± 0
cTnI (0.00–0.08 ng/ml)	0.004 ± 0.007 (*n* = 60)	0.004 ± 0.008 (*n* = 57)	0.003 ± 0.002
CD3 (690–1760/ul)	1088.3 ± 451.8 (*n* = 46)	1101.2 ± 448.3 (*n* = 45)	509 (*n* = 1)
CD4 (410–884/ul)	621.2 ± 287.1 (*n* = 46)	627.0 ± 287.6 (*n* = 45)	357 (*n* = 1)
CD8 (190–658/ul)	384.9 ± 182.1(*n* = 46)	390.4 ± 180.2 (*n* = 45)	135 (*n* = 1)
NK cells (90–536/ul)	339.4 ± 175.5(*n* = 46)	339.8 ± 177.5 (*n* = 45)	324(*n* = 1)
CD19 (90–323/ul)	211.2 ± 187.9(*n* = 46)	212.6 ± 189.8 (*n* = 45)	145(*n* = 1)

Data are expressed as mean ± standard deviation

*Abbreviation*: *ALT* the Alanine Aminotransferase test, *AST* the aspartate aminotransferase test, *ALP* the alkaline phosphatase test, *GGT* the gamma-glutamyl transpeptidase test, *ALB* the albumin test, *TP* the total protein test, *GLO* Globulin test, *A/G* the albumin globulin ratio, *TBIL* the total bilirubin test, *IBIL* the indirect bilirubin, *DBIL* the direct bilirubin test, *TBA* the thiobarbituric acid test, *PALB* the partner and localizer of BRCA2, *CHE* the cholinesterase test, GLU the blood glucose test, *BUN* the blood urea nitrogen test, *CREA* the creatinine blood test, *K*^*+*^ the potassium blood test, *Na*^*+*^ the sodium blood test, *CK* the creatine kinase blood test, *CK-MB* creatine kinase myocardial band, *LDH* the lactate dehydrogenase, *TG* Triglycerides, *CHO* Cholesterol, *HDL-C* High-density lipoprotein cholesterol, *LDL-C* the low-density lipoprotein cholesterol test, *CRP* C-reactive protein, *PT* the prothrombin time, *PTA* the pure-tone audiogram, *INR* International normalized ratio test, *aPTT* the activated partial thromboplastin time, *FIB* (fibrinogen) *CT* clotting time, *WBC* white blood cell, *RBC* red blood cell, *Hgb* the hemoglobin, *HCT* the hematocrit blood test, *pO*_*2*_ Partial Pressure of Oxygen, *pCO*_*2*_ the partial pressure of carbon dioxide, *sO*_*2*_ oxygen saturation, *HCO3* bicarbonate test, *AG* the anion gap, *Lac* the lactic acid blood test, *PCT* procalcitonin, *BNP* Brain natriuretic peptide, *cTnI* the troponin test, *CD* cluster of differentiation, *NK* cells Natural killer cells, *SD* standard deviation

Note: ↑ means the test results were above normal

Laboratory parameters, including CRP, WBC, lymphocyte, alanine aminotransferase, aspartate aminotransferase, and blood potassium (K^+^), were serially obtained during the hospitalization period and compared to the patients’ baseline data (Table 3). Only WBC count fluctuated and significantly increased during the hospitalization period (Table 3).

**Table 3:** Dynamic changes of the laboratory parameters in 67 patients with COVID-19 during hospitalization

Assay (normal range)	At Admission	During Hospitalization	
		Second test	Third test
WBC (3.5–9.5 *10^9/L)	4.7 ± 1.8	5.1 ± 1.45	5.6 ± 1.8*
Lymphocyte (1.1–3.2*10^9/L)	1.4 ± 0.6	1.5 ± 0.6	1.5 ± 0.4
ALT (9–50 U/L)	31.0 ± 31.6	27.8 ± 25.0	28.8 ± 22.2
AST (15–40 U/L)	29.0 ± 18.5	25.5 ± 14.5	23.8 ± 11.2
K^+^ (3.5–5.3 mmol/L)	3.7 ± 0.4	3.6 ± 0.4	3.7 ± 0.3
CRP (0–6 mg/L)	12.1 ± 19.3	11.9 ± 20.6	10.0 ± 16.7

Data are expressed as mean ± standard deviation

*Abbreviation*: *WBC* white blood cell, *ALT* the Alanine Aminotransferase test, *AST* the aspartate aminotransferase test, *K+* the potassium blood test, *CRP* C-reactive protein

* *p* < 0.05, compared between the third test and at admission

### Transmission of SARS-CoV-2

**Fig. 1:**
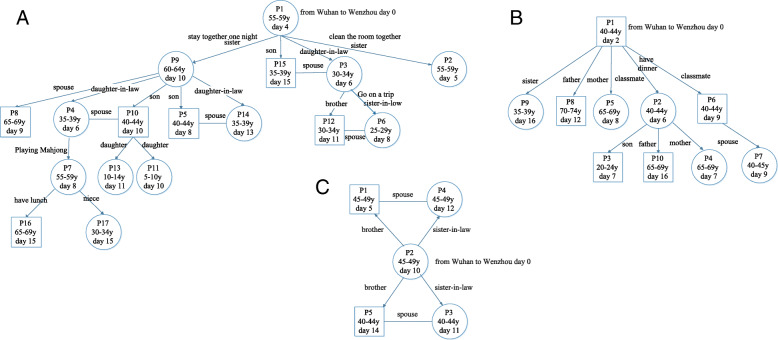
The transmission diagrams of SARS-CoV-2 for three super spreaders, **a**, **b**, and **c**. Patients numbers were coded based on the date of the onset

All patients were traced for the potential source of SARS-CoV-2 infection. Four (6.0%) of the 67 patients were identified to be the source that transmitted SARS-CoV-2 to four contacts who, in turn, transmitted the virus to other close contacts. Among the 31 patients who returned to Ruian from Wuhan, three patients directly or indirectly transmitted the infection to 16 patients, nine patients and four patients, respectively (Fig. 1). An additional three patients who returned to Ruian from other cities of Hubei Province did not transmit the infection to anyone else. Among the four local patients, there was one case without a clear source of infection, and three patients were infected by individuals from Wuhan. However, none of the four local patients transmitted the infection to others.

### Radiographic findings

**Fig. 2:**
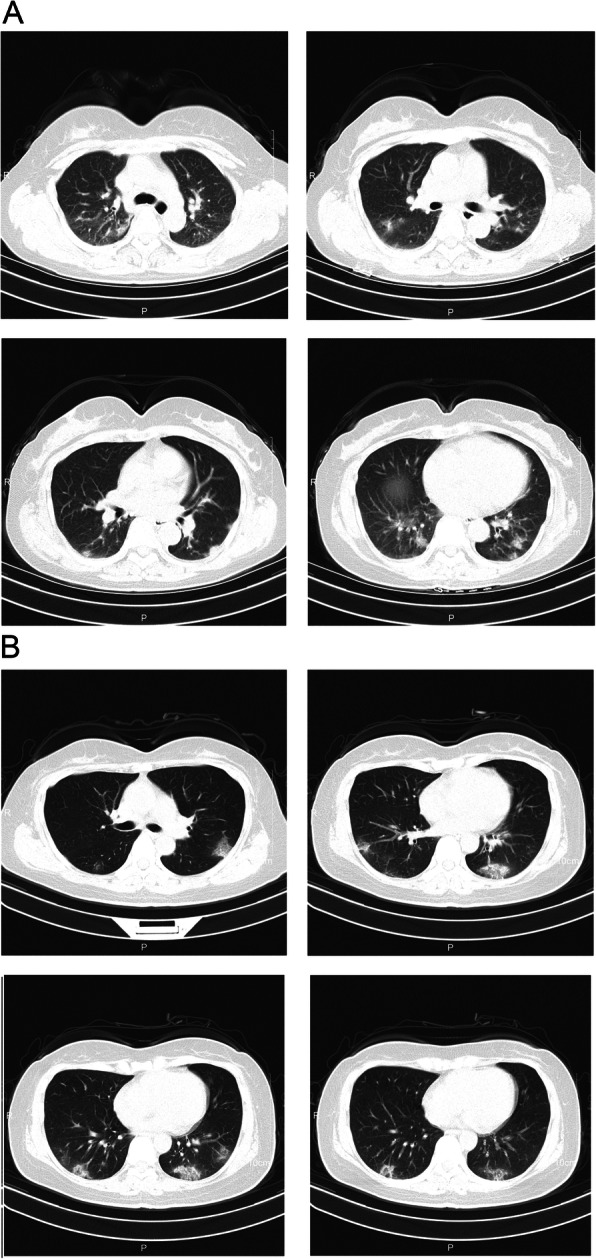
Transverse chest computed tomography (CT) images. **a**, The CT images from a woman (age range: 55–59 years old) at admission show bilateral ground-glass opacities with blurred borders; **b**, The CT images from a woman (age range: 45–49 years old) at admission shows multiple patchy ground-glass shadows in both lungs, predominately in the lower lobes, with signs of anti-halo

**Fig. 3:**
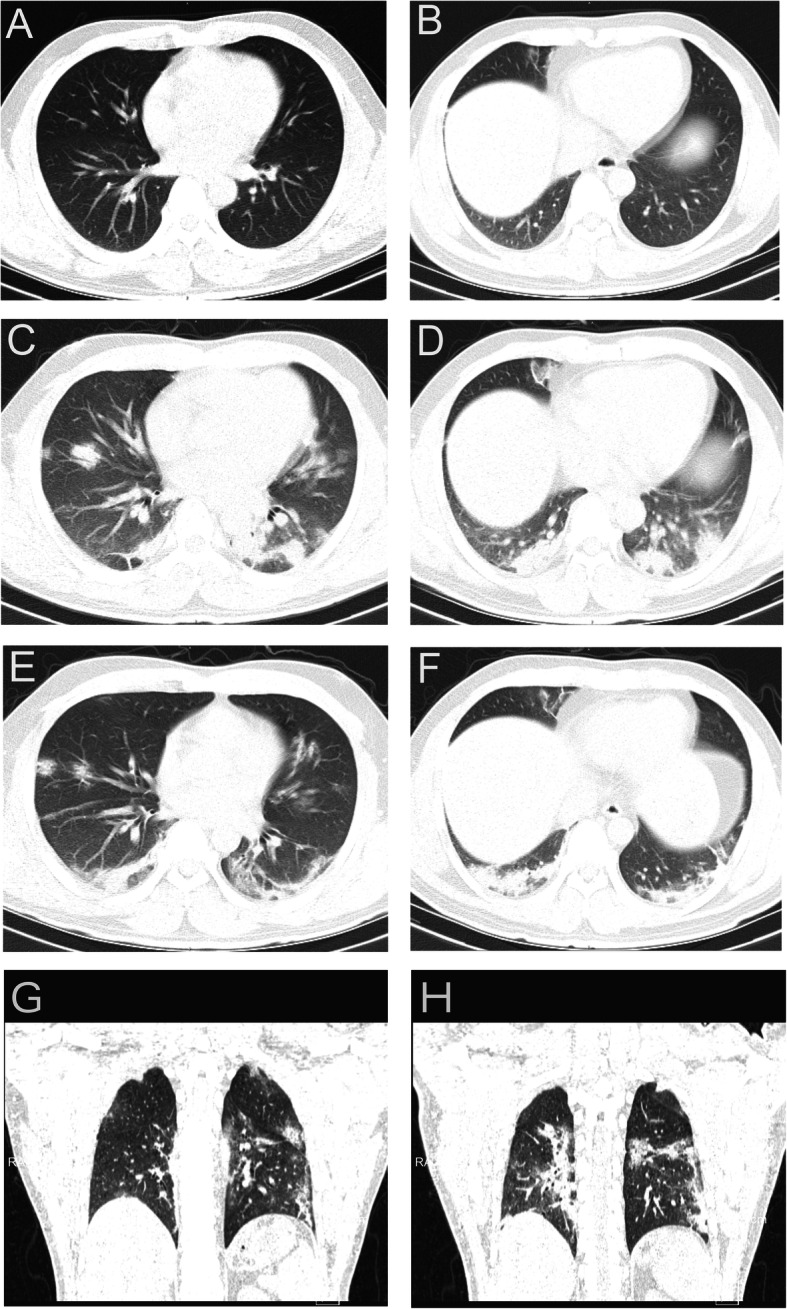
Chest computed tomography (CT) images from a man (age range: 40–44 years old) with severe disease progression. **a** & **b**), at admission, transverse chest CT images show small ground-glass opacities in the left lower lobe and high-density shadows with blurred edges; **c** & **d**, In the observation ward the day before admission, transverse chest CT images show multiple nodules with blurred borders, mainly distributed in the subpleural area, indicating a progression from the previous CT examination; (**e** & **h**), Transverse chest CT images after 14-days of treatment and three negative nucleic acid tests show increased bilateral multiple patches and stripes with blurred borders, with the prominent distribution in the subpleural area, and apparent fibrous foci

The chest CT radiographs at admission showed abnormal findings in 54 (80.6%) of the 67 patients with peripheral-zone involvement in 40 (59.7%) patients, and middle and lower lobe involvement in 31 (46.3%) patients. Bilateral involvement or multifocal lesions were identified in 48 (88.9%) patients (Fig. 2). Of the 54 patients with abnormal findings, the most common infiltration patterns were focal consolidation (*n* = 41, 75.9%) and ground-glass infiltrates (*n* = 13, 24.1%). Five (38.5%) of the 13 cases with a normal CT at admission demonstrated abnormal finding of subsequent CT examinations. Overall, abnormal findings on CT examinations progressed in 20 (29.9%) patients, including the three severe cases, during the hospitalization period (Fig. 3).

### Treatment

Antiviral treatment was based on the fourth edition of Diagnosis and Treatment program for Pneumonia Caused by COVID-19 by the National Health Commission of the People’s Republic of China (http://www.gov.cn/zhengce/zhengceku/2020-01/28/content_5472673.htm). All 67 patients received antiviral drug therapy at the first day of hospitalization. Kaletra® (lopinavir/ritonavir) (400 mg bid) was used in 62 (92.5%) patients, umifenovir (200 mg tid) in 48 (71.6%) patients, aerosolized interferon-α (500 mIU bid) in 63 (94%) patients, and oseltamivir (75 mg bid) in one (1.5%) patient until the nucleic-acid test became negative. Only one patient had an adverse event of liver dysfunction. Oxygen therapy (3 L/min) was performed in 41 (61.2%) patients until symptoms improved. No patients received mechanical ventilation. Of the three severe cases, two (66.7%) received glucocorticoid therapy. One (33.3%) patient was treated with methylprednisolone due to progression on subsequent CT examinations and worsening hypoxemia. The dose of methylprednisolone injection was 80 mg for three days, then 40 mg for two days, and finally 20 mg for one day. The other severe case received methylprednisolone 120 mg for one day and was later transferred to the First Affiliated Hospital of Wenzhou Medical University for further treatment. Immunoglobulin and plasma were not used in all patients. Only eight (11.9%) patients with secondary bacterial infections were treated with antibiotics treatment (Table 4).

**Table 4:** Treatment of 67 patients with COVID-19

Treatment	All patients (***n*** = 67)	Common patients (***n*** = 64)	Severe patients (***n*** = 3)
**Oxygen therapy**	41 (61.2)	39 (60.9)	3 (100)
**Mechanical ventilation**	0	0	0
**CRRT**	0	0	0
**ECMO**	0	0	0
**Antibiotics**	8 (11.9)	7 (10.9)	1 (33.3)
**Antifungals**	0	0	0
**Antiviral drug**			
KALETRA (Lopinavir / Ritonavir)	62 (92.5)	59 (92.2)	3 (100)
Umifenovir	48 (71.6)	46 (71.8)	2 (66.7)
Aerosolized α interferon	66 (98.5)	63 (98.4)	3 (100)
Oseltamivir	1 (1.5)	1 (1.6)	0
**Glucocorticoid therapy**	2 (3.0)	0	2 (66.7)

Data are expressed as number (%)

*Abbreviation*: *CRRT* Continuous Renal Replacement Therapies, *ECMO* Extracorporeal membrane oxygenation

### Short-term outcomes

By February 17, 2020, there were no deaths among the 67 patients and no detected infection among medical staff in the department. Fifty-four (80.6%) patients completely recovered and had a negative nucleic acid test. These patients were discharged from the hospital and their average hospitalization stay was 9.0 ± 3.0 days. Other patients, including the three severe cases, were improving by February 17, 2020, and all had a negative nucleic acid test at the time of discharge.

By March 3, 2020, all 67 patients had completely recovered and discharged with negative nucleic acid tests. The patients were transferred to a medical observation isolation center for further observation. During this period of observation, 14 (20.9%) patients developed a positive nucleic acid test; however, all patients eventually tested negative by March 20, 2020.

### Positive predictors of pneumonia

According to univariate analyses, independent predictors for pneumonia included age, cough, sore throat, productive cough, and CRP level. However, no independent predictor was found for pneumonia using multiple regression (See Table 5).

**Table 5:** Predictors of pneumonia according to data of 67 patients with COVID-19

	Simple logistic regression Odds ratio (***p*** value)	Multiple logistic regression Odds ratio (***p*** value)
**Age**	1.06 (*p* = 0.03)	1.07 (*p* = 0.39)
**Gender**	4.96 (*p* = 0.06)	9.52 (*p* = 0.18)
**Exposure history**	1.70 (*p* = 0.71)	
**Incubation**	1.12 (*p* = 0.88)	
**Period from onset to admission**	0.92 (*p* = 0.59)	
**Cardiovascular disease**	1	
**Digestive disease**	1	
**Cancer**	1	
**Nervous system disease**	1	
**Respiratory disease**	1	
**Kidney disease**	1	
**Hypertension**	0.64 (*p* = 0.62)	
**Diabetes**	0.92 (*p* = 0.94)	
**Connective tissue disease**	1	
**Pregnancy**	1	
**Drinking**	1	
**Smoking**	0.62 (*p* = 0.46)	
**Fever**	4.33 (*p* = 0.07)	29.72 (*p* = 0.29)
**Headache**	1	
**Dizziness**	1	
**Muscular soreness**	1	
**Running nose**	1	
**Sore throat**	6.44 (*p* < 0.001)	1
**Dry cough**	6.00 (*p* = 0.02)	283.07 (*p* = 0.19)
**Productive cough**	6.44 (*p* < 0.001)	0.15 (*p* = 0.47)
**Chest distress**	1	
**Shortness of breath**	1	
**Abdominal pain**	1	
**Diarrhea**	0.59 (*p* = 0.66)	
**Nausea or vomiting**	1	
Lymphocyte (1.1–3.2*10^9/L)	0.24 (*p* = 0.008)	
**ALT (9–50 U/L)**	1.09 (*p* = 0.11)	
**CRP (0–6 mg/L)**	12.09 (*p* = 0.03)	200.82 (*p* = 0.10)

*Abbreviation*: *ALT* the Alanine Aminotransferase test, *CRP* C-reactive protein

## Discussion

The present study outlines the current first-line data, including clinical and radiographic features, treatment, and short-term outcomes of COVID-19 patients in the Wenzhou area, which is outside of Wuhan. Of the 67 COVID-19 cases admitted to our department from January 21 to February 7, 2020, only three were severe cases and 64 were common cases. On February 17, 2020, 54 patients had completely recovered and discharged from the hospital, and the remaining cases, including the three severe cases, were improving. Importantly, based on the transmission patterns among the included patients and recently reported cases, SARS-CoV-2 infection was shown to be highly contagious [6, 10–12].

In the present study, more than half (53.7%) of patients were male and a high proportion of the patients were 19–45 (49.3%) and 46–65 (32.8%) years old. Due to the small sample size of the present study, these findings may be not clinically significant; however, several other studies in China, Europe and the United States have demonstrated similar findings [3, 13, 14]. In the present study, two of three severe cases were male, similar to the observation in the study by Chen et al. [3]. This suggests that male patients may have a worse prognosis than females; however, more evidence from larger studies is needed to confirm this hypothesis.

Only one child (5 years old) and no very elderly patients (> 80 years) were included in the present study. One possible reason is that young and middle-aged adults have more social activities, and thus increased the opportunity to be exposed to infectious sources. Compared to the first two clinical studies describing COVID-19 patients in the Wuhan area, the age of our patients was younger [3, 4]. Nevertheless, the 19–65 age group is important, and although most cases in this age group are mild, they may act as spreading sources.

The top three symptoms in the present study were fever (86.6%), cough (77.6%), and productive cough (52.2%), which is consistent with the findings of the first recent reports on patients with COVID-19 infection [3, 4]. The rate of diarrhea in our cohort was 7.5%, which is higher than previously (2–3%) reported in COVID-19 patients [3, 4], but lower (23.6–41.7%) compared to patients with SARS-CoV-1 pneumonia, which outbroke 17 years ago [15, 16]. It has been reported that viral infection can affect the gastrointestinal tract [17–19]. In addition, recent data suggest that COVID-19 may be able to directly enter gastrointestinal enterocytes via ACE2 receptors [20, 21]. The high rate of diarrhea in COVID-19 is an important clinical observation, considering the large population affected by the disease worldwide. Thus, clinical providers should keep this symptom in mind in the diagnosis and treatment of COVID-19. However, whether diarrhea is related to epithelial cell receptors in the respiratory tract and gut, as it is with H5N1 avian influenza [22], or effects clinical outcomes is unknown and requires further research.

In the present study, there were only three (4.5%) severe cases, which was much less than that reported previously [3, 4]. We found that exposure to Wuhan patients, connective tissue disease, undergoing immunosuppressive therapy, chest distress, shortness of breath, and hypoxemia were associated with severity of the disease. However, since there were only three severe cases in the present study, these findings need to be interpreted with caution.

In the present study, leukopenia, abnormal neutrophil count, lymphopenia, and thrombocytopenia were observed in 25.4, 28.3, 17.9 and 11.9% of patients, respectively, at admission. These findings suggest that a low WBC count may be a clinical feature of COVID-19 infection [23], although the WBC count is normal in most of the patients infected with SAS-CoV-2. We observed that the WBC count increased modestly in some COVID-19 patients during the advanced stage of the disease. This observation is consistent with the study by Chen et al. which reported that 24% of their 99 Wuhan patients had increased leukocyte levels [3]. However, the underlying mechanisms for an increased WBC count during the advanced stage of the disease needs to be further investigated. Another apparent characteristic of COVID-19 patients is that the initial fever was not very high (37.3 ± 0.8 °C), regardless of the severity of the disease, suggesting that although fever was the most frequent symptom, some patients with normal temperature should not be ignored.

CRP is known as a common laboratory marker of systemic inflammation, and generally CRP levels are lower in viral infections than in bacterial infections [24]. In the present study, 47.8% of patients had increased CRP levels, which is consistent with the study from Chen et al., reporting that CRP was increased in 86% of patients [3]. Other studies also reported elevated levels of CRP in COVID-19 patients in Wuhan [3, 13, 25]. Therefore, elevated CRP could provide additional laboratory investigational data for COVID-19 patients, particularly in the absence of specific COVID-19 testing. Compared to the common cases, the severe cases had significantly increased levels of GGT, CK, LDH and aPTT. Therefore, these biomarkers may be useful for predicting the severity of the disease; however, larger studies are required to further explore this hypothesis.

Typical chest CT imaging findings have been shown to be accurate to diagnose SARS-CoV-2 pneumonia [26–30]. Unlike the previous two studies on Wuhan patients, [3, 4] where all of the patients were diagnosed by radiography, 13 (19.3%) cases in our cohort had a normal chest CT examination, and the CT imaging remained normal for eight of these patients during hospitalization.

Our study had less severe cases compared to previous studies. This may have been due to the rigorous screening method in the Ruian area with almost every patient’s contacts screened. The high mortality of COVID-19 worldwide (estimate mean of 5.7% globally [31]) may be because the prevalence of COVID-19 is much higher than the number of cases detected among the population.

In the present study, patients were treated mainly by oxygen therapy and antiviral drugs. Only two severe cases were given glucocorticoid therapy. This management is different from that for patients infected with SARS-CoV-1, for whom corticosteroid therapy was performed [15]. Compared to the reported rate of patients receiving ICU care (26%) and mortality (4.3%) in Wuhan [13], no patients were admitted to the ICU and the mortality rate was 0% in our study. This is consistent with other reports outside Wuhan, China, and abroad [32, 33]. We speculate that this may be related to multiple reasons. 1) Although the time from symptom onset to hospitalization in our study was 3.8 ± 2.3 days, patients were usually screened and treated as “suspected patients” in the observation ward 1–2 days before admission to hospital. Therefore, our patients generally received rest, symptomatic therapy, and oxygen within three days of symptom onset, likely improving prognosis. Patients also received antiviral therapy earlier, although there is no discrete evidence at this time that antiviral therapy is efficacious for treating COVID-19. 2) In the present study, severity of disease was associated with exposure to patients from Wuhan, suggesting that with passage, virulence and pathogenicity of the virus gradually decreases; however, further studies are required to verify this observation.

According to our regression analysis, we did not find any independent predictor for pneumonia in COVID-19 patients. However, some demographic characteristics, symptoms, and laboratory parameters such as age, cough, sore throat, and CRP level can be used as a reference by clinicians to diagnose pneumonia.

To our best knowledge, this is the first clinical report on COVID-19 outside the Wuhan area. Wenzhou is the most affected area outside of Hubei Province, and the patients in this area would be of high representativity for those outside the Wuhan area. A limitation of the study is the unavoidable bias due to the small sample size, and thus some findings of the present study may not have clinical significance. A further collection of related data is needed to confirm our findings.

## Conclusions

COVID-19 is an emerging and highly infectious viral disease. Cases in the Wenzhou area are milder, with better prognosis, compared to those in Wuhan, China. Timely and appropriate screening, diagnosis, and treatment are the key to achieve good outcomes.

## Data Availability

The datasets used and/or analyzed during the current study are available from the corresponding author on reasonable request.

## Abbreviations

COVID-19: Coronavirus disease 2019
CT: Computerized tomography
SARS-CoV-2: Severe acute respiratory syndrome coronavirus 2
WBC: White blood cell
SpO2: Peripheral capillary oxygen saturation
PaO2: Partial pressure of oxygen
FiO2: Fraction of inspired oxygen
sO2: Oxygen saturation
CDC: Centers for Disease Control
SD: Standard deviation
CRP: C-reactive protein
GGT: Gamma-glutamyl transpeptidase
LDH: Lactate dehydrogenase
CK: Creatine kinase
aPTT: Activated partial thromboplastin time
ALT: Alanine aminotransferase
AST: Aspartate aminotransferase
